# The Prevalence of Incidental Findings at Cardiac MRI

**DOI:** 10.2174/1874192400802010020

**Published:** 2008-04-02

**Authors:** David A McKenna, Monish Laxpati, Patrick M Colletti

**Affiliations:** Department of Radiology, Keck School of Medicine, University of Southern California, USA

**Keywords:** Cardiac MRI, incidental findings

## Abstract

**Object or purpose of study:**

As the field of view of cardiac magnetic resonance imaging (CMR) includes the thorax and upper abdomen, it is not surprising that these studies can reveal incidental extra-cardiac abnormalities. The purpose of this study is to determine the prevalence of these incidental findings.

**Materials, Methods and Procedures:**

132 volunteer participants with a mean age of 74.2 years (range, 61-89 years; 127 males and 5 females) had CMR with 7 sequences. All images were retrospectively reviewed by a radiologist, specifically assessing for non-cardiac findings. Visualized abnormalities were noted and categorized according to significance. Clinically significant findings were defined as those requiring further clinical or radiological work-up, with moderately significant findings defined as those that may affect patient care depending on medical history or symptoms. Remaining findings were considered clinically insignificant.

**Results:**

Within the group, 107 participants (81%) had extra-cardiac findings, with 63 (48%) having multiple findings. A total of 224 incidental findings were visualized, with at least one clinically significant and moderately significant finding found in 23 (17%) and 43 (33 %) of the subjects, respectively. Potentially clinically significant findings included pulmonary nodules, solid or complex lesions of the solid abdominal viscera and thyroid, and aortic pathology including aneurysm. The most prevalent incidental findings were however benign appearing, including renal and hepatic cysts, hemangiomas, and atelectasis. The SSFP coronal localizer, SSFP axial localizer, and short axis SSFP cine oblique sequences were most sensitive at detecting incidental findings (p = 0.013 vs four other sequences) with 47%, 46%, and 41% detection respectively, with no significant difference between these three multislice sequences (p = 0.369).

**Significance of the conclusions:**

In total, 81% of our volunteers had extra-cardiac findings, of which 17% were potentially clinically significant, necessitating further work up. We believe that these numbers appear high compared to prior similar studies performed at Cardiac CT. This may be related to the relatively older cohort examined here. In conclusion it is important to look beyond the heart when reviewing cardiac MRI studies and carefully assess the entire field of view for abnormalities.

## INTRODUCTION

In addition to evaluating the heart and great vessels, the field of view (FOV) of cardiac magnetic resonance (CMR) includes the thorax and upper abdomen. CMR scans can therefore detect findings that are incidental to the initial indication that an examination was requested. The detection and characterization of these non-cardiac findings represents a challenge to the interpreting physician. This article reviews the existing literature concerning the incidence and treatment of incidental findings at cardiac CT and recommends a methodical approach to evaluating similar findings with CMR.

As CMR studies may not be over-read by radiologists, it is especially important for reviewing physicians to be mindful of incidental findings. These findings may be clinically significant, requiring further work-up or treatment.

One review of cardiac CT reports demonstrated 323 extra-cardiac findings in 291 patients (30.2%). Esophageal cancers, lung cancers, and aortic aneurysms were the most clinically serious findings [1]. A limitation of their study was that they only reviewed radiology reports, and may therefore have underestimated the true incidence of extra cardiac pathology. Our aim was to evaluate how often CMR could detect incidental pathology and to try to quantify the prevalence and characterization of incidental extra cardiac findings in a screening population. As the first published study evaluating incidental findings at CMR we also aim to suggest a methodical approach to evaluating these findings.

## METHODS

### Subjects

Participants in this University of Southern California Institutional Review Board approved, HIPAA compliant investigation had a CMR exam using a standard study protocol between the dates of November 23, 2005 and July 20, 2006. Each study was composed of 7 individual sequences, including axial and coronal localizer, vertical long axis, short axis, mitral valve, aortic views, and black blood views. All images were retrospectively reviewed to specifically assess for incidental findings. The mean age of these patients was 74.2 years (range, 61-89 years). The study group was composed of 127 males and 5 females All of the visualized extra-cardiac findings were noted and categorized according to clinical significance. Clinically significant abnormalities were defined as those requiring further clinical or radiological work-up, with intermediate significance defined as those that may affect patient care depending on medical history or current symptoms. Remaining incidental findings were considered benign.

### MR Imaging Technique

CMR studies were obtained with a 1.5-Tesla whole body scanner (General Electric, Milwaukee, WI), with 11.1 software with a cardiac phased array coil and cardiac gating. Heart rate and peripheral blood pressure were recorded before and after each exam. Coronal and axial steady state free precession SSFP localizers were obtained with a TR/TE; 3.6/ 1.6, single echo with a bandwidth of 125kHz, and 8mm slice thickness with no inter-slice gap. The acquisition matrix was 224x 224 with a single excitation and respective field of views of 35 x 35cm for coronal images and 35 x 26.5cm for axial images.

Contiguous breath-hold, EKG triggered 8 mm thick single slice vertical long axis SSFP cine and short axis, 2D SSFP, TR 3.6 msec, TE 1.6 msec, flip angle 45 degrees, with a field of view of 44 cm and a 224x224 matrix, 20 phase intervals over the cardiac cycle, cine images were acquired from the apex to the base of the left ventricle.

A single slice breath held 2D PC Cine VENC 200, 2 cm below mitral valve plane parallel to MV was acquired to evaluate trans-mitral flow. A single slice SSPP cine was acquired in the axial plane at level of the main pulmonary artery. At this same level, 4 double-inversion black-blood axial 8 mm thick images were acquired.

### MR Image Interpretation

A single reader (Board eligible radiologist with a 1 year experience in the interpretation of CMR imaging) retrospectively reviewed all CMR images at an independent workstation (Advantage Windows 4.0 General Electric, Milwaukee, WI). All of the CMR scans were specifically analyzed for extra cardiac pathology. The interpreting radiologist was blinded to patient’s clinical history. It should be noted that strict objective criteria for incidental pathology identification have not been reported, and reader diagnosis was therefore necessarily somewhat subjective. Each of the sequences, including the axial and coronal localizers, was carefully scrutinized with incidental findings categorized as either clinically significant or insignificant. Significant findings were defined as those requiring further diagnostic work-up or some type of medical or surgical intervention. Patients with clinically significant findings were notified. However, clinically insignificant findings were not reported to patients or their physicians. The CMR exams in this study were performed for research purposes only, and no official report was generated.

## RESULTS

**Fig. (1):**
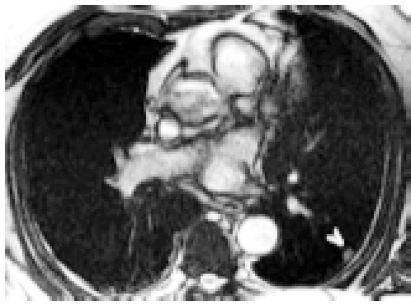
SSFP axial localizer demonstrates a 1 cm peripheral pulmonary nodule (arrowhead). Follow-up revealed no change over 6 months in this 81 year old man.

**Fig. (2):**
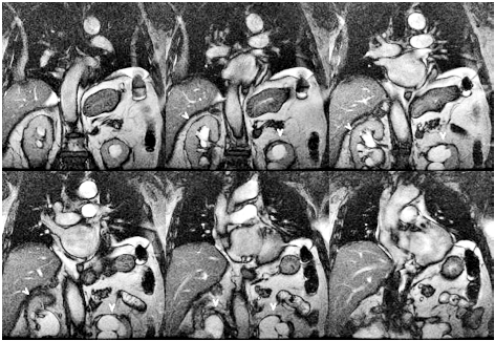
SSFP Coronal localizer demonstrates bilateral hydronephrosis (arrowheads) in this 87 year old man.

**Fig. (3):**
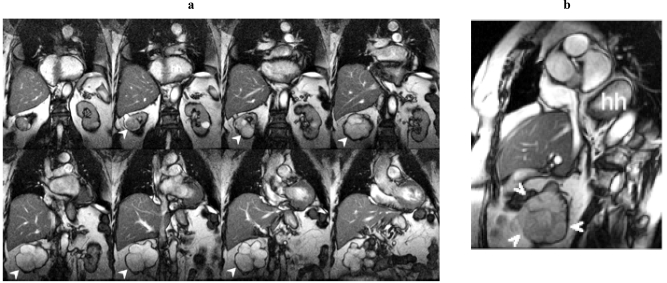
**a.** SSFP coronal localizer. Right renal solid tumor (arrowheads). Large hiatus hernia (hh). Incidental renal cell carcinoma in this 84 year old woman. **b.** Short axis SSFP cine frame. Right renal solid tumor (arrowheads). Large hiatus hernia (hh). Incidental renal cell carcinoma in this 84 year old woman.

**Fig. (4):**
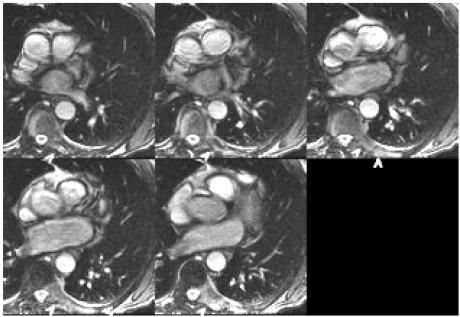
SSFP axial localizer: thoracic spine vertebral body, left posterior element, and left rib tumor (arrowheads). Metastatic prostatic cancer in this 83 year old man three years after initial prostate resection.

**Fig. (5):**
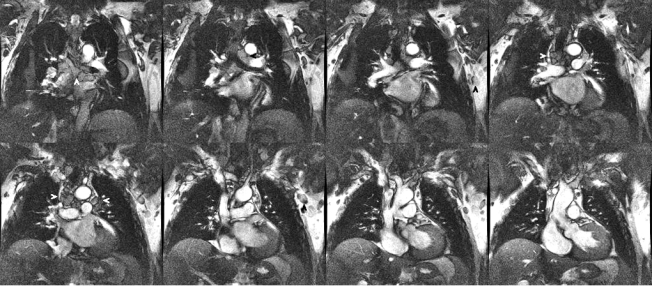
SSFP coronal localizer: multiple enlarged paratracheal, aorto-pulmonary window, coronal, Hilar, and axillary lymph nodes (arrowheads). 70 year old man with sarcoidosis.

Descriptive statistics of mean and range were used to summarize the patient cohort with respect to clinical, MR imaging, and outcome variables. Of the 132 participants, 107 (81 %) had at least one extra cardiac finding, with 48% having multiple findings. A total of 224 incidental findings were visualized, including 25 potentially significant findings and 50 findings of intermediate importance (Table **1a**- **1c**, Figs. **1-5**). The most common significant finding was a pulmonary nodule (5 patients) with the commonest intermediate finding being aortic plaque seen in 20 patients. Significant findings included pulmonary or pleural based nodules, solid or complex renal masses and liver lesions as well as aortic aneurysms. Most incidental findings were likely clinically insignificant; of these, renal cysts, hepatic cysts and hemangiomas, and atelectasis were the most prevalent. The frequency of visualization of extra-cardiac pathology on the various sequences was also compared. In our study, the coronal localizer, axial localizer, and short axis multislice views were the best at detecting incidental findings compared with the single slice VLA cine, trans-mitral flow cine, aortic pulsatility cine, and the 4 slice aorta black blood sequence (p = 0.013). With 47%, 46%, and 41% of all findings visualized on the coronal localizer, axial localizer, and short axis multislice views, respectively, there was no significant difference between these three sequences (p= 0.369) (Table **2**).

## DISCUSSION

While CMR studies are generally conducted on individuals who are either symptomatic or at high-risk for cardiovascular disease with the purpose of visualizing the heart, inclusion of the surrounding structures is unavoidable. Although many of the incidental findings will turn out to be benign after further work-up, other significant pathologies may be detected early enough to affect outcome for the patient. The detection and characterization of these non-cardiac findings represents a challenge to the physicians who are reviewing and reporting these studies.

This study demonstrates that CMR may uncover a large number of incidental non-cardiac findings and therefore the physicians interpreting CMR exams must judiciously evaluate all structures within the FOV. Although the majority of these findings tend to be benign in nature, some will be clinically significant, requiring further examinations or treatments. Incidental findings at cardiac CT appear frequent with some studies suggesting the incidence of non-cardiac findings at coronary CTA between 25% to 61% of cases with 5% to 10% considered major [2]. According to our study 17% of patients undergoing a screening CMR will have potentially significant incidental findings.

Although no prior studies on the prevalence of incidental non-cardiac findings in CMR have been published to date, several studies have looked at this issue in regards to other cardiac imaging modalities. A study by Horton *et al. *looked at 1326 screening electron-beam computed tomography (EBCT) studies and found that 7.8% had significant extra cardiac pathology [3]. In a similar prior study of 1812 EBCT exams, Hunold *et al. *found that 53% of the patients had visible non-coronary pathology, including cardiac abnormalities [4]. However, 67% of these abnormalities were extra cardiac in origin. While these findings include a number or minor, clinically insignificant abnormalities, 9.3% of the 2055 total extra coronary findings required further diagnostic testing [4].

Shih *et al. *reviewed 566 ^99m^Tc tetrofosmin gated cardiac SPECT exams looking specifically for abdominal abnormalities. They found 234 abnormalities, including mostly abnormal bone marrow uptake of the vertebrae and duodeno-gastric and entero-gastric reflux [5]. Overall, these studies emphasize the importance of examining the entire FOV for incidental findings on cardiac imaging studies.

Some imaging centers limit the cardiac imaging field-of-view (FOV) as much as possible to include only the heart and coronary arteries. The American College of Cardiology Task Force for Training in Computed Tomography states “During a cardiac CT examination, the standard use of a small field-of-view (e.g., limited lung fields) precludes a complete evaluation of the entire thorax. The patient and the referring physician should understand that the focus of the cardiac CT examination is the detection of cardiac disease, and the scan does not encompass the entire lung field [6]”. However given that patients sometimes present with vague symptoms CMR can facilitate the detection alternative pathology or concurrent pathology and therefore to make alternative diagnoses.

Despite the fact that institutions each have different standard protocols for CMR and may perform different types of sequences, it seems reasonable that the types and incidence rates of extra-cardiac pathologies would be similar. This study demonstrates the importance of reviewing the localizer sequences, as it appears that incidental findings are more commonly identified on the localizer sequences (p= 0.319). This may be secondary to the fact that localizer sequences have a larger FOV and therefore are more likely to encompass extra-cardiac pathology. We used a SSFP sequence for localization, which is fast and heavily T2 weighted. The identification of pathology on these sequences alone is somewhat limited as these sequences are not contrast enhanced and therefore may not truly characterize the lesions. Correlation with prior history and imaging is important to further characterize the significance of these findings and reduce the risk of false positive studies.

This study has several limitations. While the study population depicts a fairly typical patient populace who may undergo a screening CMR study, with a moderate cardiac risk profile, it was not composed of an actual sample of diagnostic exams. Our study population is composed of nearly all males (96%). Although many of these incidental findings would be expected to occur in similar frequencies in females, actual rates may be found to be somewhat different. For example, female patients would be expected to have a larger number of incidentally discovered breast masses. Our study population had a mean age of 74.2 years, which may be older than the mean age of patients who typically undergo screening CMR exams. It can be expected that the prevalence of incidental findings increases with age, as was demonstrated with EBCT [4]. Finally, this study is limited by the lack of follow-up on the patients. While patients with clinically significant findings were notified, further information regarding further diagnostic testing and treatment is unavailable. However, the primary goal of this study was to determine the prevalence and significance of these abnormalities and not to follow their outcome.

## CONCLUSION

The purpose of this study was to determine the prevalence of incidental findings seen at screening CMR studies. In total, 81% of our volunteers had extra-cardiac findings, of which 17% were potentially clinically significant, necessitating further work up. With incidental findings were best visualized on localizer sequences. Despite the fact that the majority of our findings were benign and that current practice regarding the reporting of incidental findings varies among institutions our study confirms that it is important to look beyond the heart when reviewing CMR studies and carefully assess the entire field of view for abnormalities.

## Figures and Tables

**Table 1a.** Clinically Significant Findings

**Finding**	**Findings Identified**	**Participants with Finding**	
Pulmonary Nodule	7	5	(3.8%)
Solid Renal Mass or Complex Cyst	3	3	(2.3%)
Non-Cystic Liver Lesion	3	3	(2.3%)
Pleural Nodule	3	3	(2.3%)
Abdominal Aortic Aneurysm	2	2	(1.5%)
Vertebral Lesion	1	1	(0.8%)
Ascending Aortic Aneurysm	1	1	(0.8%)
Pericardial Effusion	1	1	(0.8%)
Loculated Ascites	1	1	(0.8%)
Hydronephrosis	1	1	(0.8%)
Aortic Dissection	1	1	(0.8%)
Non-cystic Thyroid Nodule	1	1	(0.8%)
Total	25	23	(17.4)

**Table 1b.** Intermediate Significant Findings

**Finding**	**Incidence**	
Aortic Plaque	20	15.20%
Pleural Effusion	12	9.10%
Hiatal Hernia	8	6.10%
Gallstones	7	5.30%
Renal AML	1	0.80%
Duplex Collecting System	1	0.80%
Atrophic Kidney	1	0.80%

**Table 1c.** Clinically Insignificant Findings

**Finding**	**Number of Findings**	**Number of Patients**	**% of Patients**
Renal Cyst	55	40	30.3%
Hepatic Cyst vs. Hemangioma	35	28	21.2%
Atelectasis	20	20	15.2%
Mediastinal Node	6	6	4.5%
Sternotomy Wires/Clip	6	6	4.5%
Spinal Hemangioma	7	6	4.5%
Diverticulosis	4	4	3.0%
Pleural Thickening	3	3	2.3%
Splenic Cyst	2	2	1.5%
Degenerative Disc Disease	2	2	1.5%
Pulmonary Scar	2	2	1.5%
Scoliosis	1	1	0.8%
Paraspinal Cyst	1	1	0.8%
Lipoma	1	1	0.8%
Calyceal Diverticulum	1	1	0.8%
Eventration of Diaphragm	1	1	0.8%
Thyroid Cyst	1	1	0.8%
Axillary Lymph Node	1	1	0.8%

**Table 2.** 

**Sequence**	**Total Number of Findings **		**Significant Findings**	
Axial Localizer	104	46%	12	48%
Coronal Localizer	105	47%	11	44%
Short Axis	92	41%	8	32%
Black Blood	46	21%	7	28%
Aorta	19	8%	3	12%
Vertical Long Axis	13	6%	2	8%
Mitral Valve Flow	2	1%	0	0%
*All Sequences*	224	100%	25	100%
